# Contemporary Management of Benign and Malignant Parotid Tumors

**DOI:** 10.3389/fsurg.2018.00039

**Published:** 2018-05-11

**Authors:** Jovanna Thielker, Maria Grosheva, Stephan Ihrler, Andrea Wittig, Orlando Guntinas-Lichius

**Affiliations:** ^1^Department of Otorhinolaryngology, Universitätsklinikum Jena, Jena, Germany; ^2^Department of Otorhinolaryngology, Head and Neck Surgery, University of Cologne, Cologne, Germany; ^3^Laboratory for Dermatohistology and Oral Pathology, Munich, Germany; ^4^Department of Radiotherapy and Radiation Oncology, Universitätsklinikum Jena, Jena, Germany

**Keywords:** salivary gland tumor, parotid cancer, parotidectomy, facial nerve, radiotherapy, chemotherapy, targeted therapy

## Abstract

To report the standard of care, interesting new findings and controversies about the treatment of parotid tumors.

Relevant and actual studies were searched in PubMed and reviewed for diagnostics, treatment and outcome of both benign and malignant tumors.

Prospective trials are lacking due to rarity of the disease and high variety of tumor subtypes.

The establishment of reliable non-invasive diagnostics tools for the differentiation between benign and malignant tumors is desirable. Prospective studies clarifying the association between different surgical techniques for benign parotid tumors and morbidity are needed. The role of adjuvant or definitive radiotherapy in securing loco-regional control and improving survival in malignant disease is established. Prospective clinical trials addressing the role of chemotherapy/molecular targeted therapy for parotid cancer are needed. An international consensus on the classification of parotid surgery techniques would facilitate the comparison of different trials. Such efforts should lead into a clinical guideline.

## Introduction

**Figure 1:**
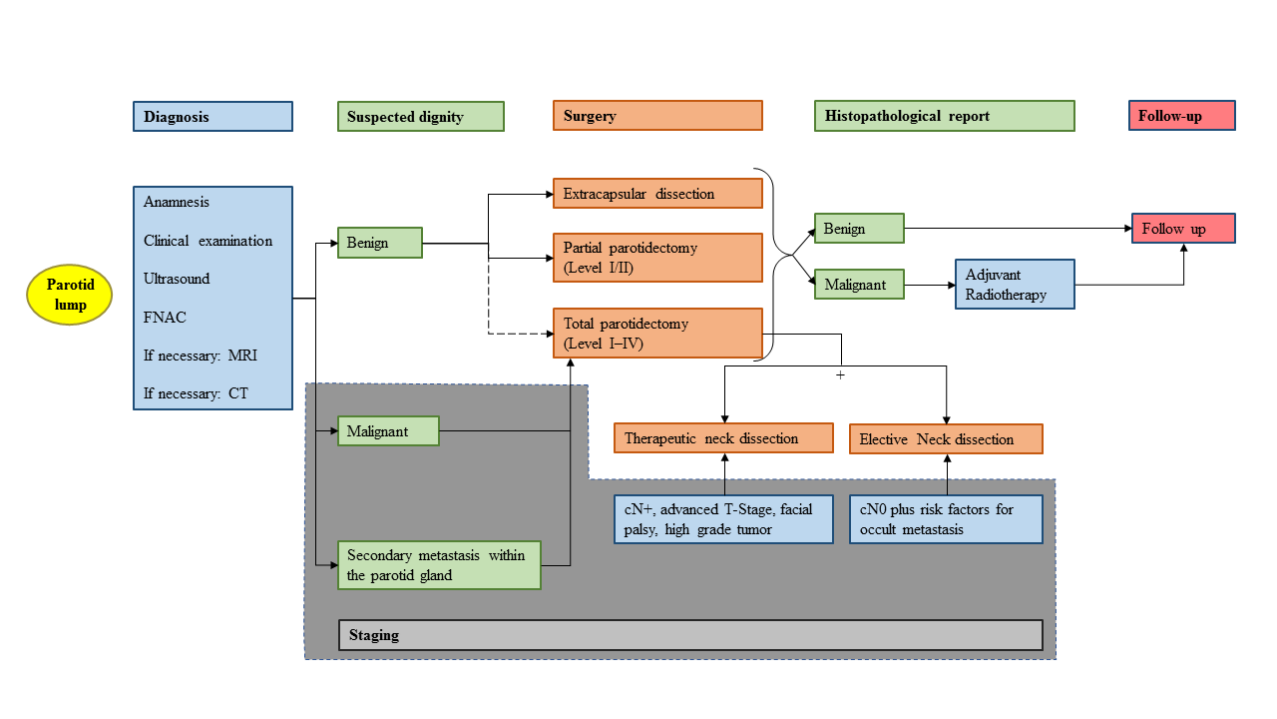
Treatment algorithm. Simplified representation of the treatment of a primary and resectable parotid lump from initial presentation, examination, diagnostics, surgery, adjuvant treatment, ending with the follow-up. This algorithm summarizes data from the studies cited in the article (mainly studies with only moderate evidence) and includes the personal experience of the authors. FNAC, fine needle aspiration cytology; MRI, magnetic resonance imaging; CT, computed tomography.

Parotid tumors are the most frequent salivary gland tumors. Histological examination of most parotid tumors proves benign disease (80%), whereas only 20% are malignant (1, 2). First sign is in most cases a parotid lump. Malignant parotid tumors can appear very similar to a benign process as they can grow slowly, displacing instead of infiltrating neighboring structures and seem to be mobile. Only about one third of the malignant tumors present initially with a facial palsy, skin infiltration, or obvious neck metastasis as clinical signs of malignancy (3, 4). Due to similar clinical and partly imaging findings we summarize the management of benign and malignant parotid tumors within one review. This review presents a straight-forward diagnostic approach to differentiate benign tumors in typical localization from deep lobe tumors and especially form malignant tumors. The up-to-date principles of these most important surgical approaches are presented. Malignant tumors often need facial nerve rehabilitation management nowadays at best performed as single-step procedures together with the oncological surgery (5, 6). Many malignant tumors need adjuvant radiotherapy (7, 8). Special situations like recurrent benign or malignant tumor and metastatic disease are also elucidated. A short summary of all important presented diagnostics and treatment and the related evidence level is given in Table 1. In addition, a personal proposal of the authors for an algorithm summarizing the treatment of a parotid lump is shown in Figure 1. Many presented aspects also apply for other major salivary tumors or minor salivary gland tumors. Nevertheless, there are several important differences not discussed in this review.

**Table 1:** Management of parotid tumors – levels of evidence.

**Step of management**	**Comment**	**Evidence level**
**Diagnostics**		
Clinical examination	Important for the differentiation between benign and malignant tumor: fast growing, facial palsy, pain, fixation are signs of malignancy	Cohort studies
Ultrasound and fine-needle aspiration cytology	Accurate for benign superficial tumors	Cohort studies, meta-analysis of cohort studies
MRI	Accurate for large tumors, deep lobe tumors, malignant tumors	Cohort studies
Core needle biopsy	Alternative if fine-needle aspiration cytology is not available of if the cytopathologist suggests that fine-needle aspiration cytology is not sufficient for diagnosis	Cohort studies
Frozen sections	Alternative if fine-needle aspiration cytology is not available or if fine-needle aspiration cytology was not conclusive	Cohort studies, meta-analysis of cohort studies
**Treatment**		
Wait-and-scan	For selected cases of Warthin tumors	Descriptive studies
Partial or superficial parotidectomy	For benign tumors in the superficial lobe	Cohort studies, meta-analysis of cohort studies
Extracapsular dissection	For selected benign tumors in the superficial lobe	Cohort studies, meta-analysis of cohort studies
Total parotidectomy	For benign tumors of the deep lobe, extension into the parapharyngeal space, malignant tumor without facial nerve infiltration	Cohort studies
Radical parotidectomy	For malignant tumor with facial nerve infiltration	Descriptive studies
Curative neck dissection	For cN +parotid cancer including level I-V	Cohort studies
Elective neck dissection	For cN +parotid cancer, at least level I-III	Cohort studies
Facial nerve rehabilitation	If reconstruction is possible in case of parotid cancer with facial nerve infiltration as single stage procedure	Descriptive studies
Radiotherapy, adjuvant	For all cases of advanced-stage disease (T3/T4), high-grade tumors, always for adenoid cystic carcinoma, close or positive margins, bone invasion, lymph node metastases (more than three metastatic nodes), perineural and/or vascular invasion	Cohort studies
Radiotherapy, definitive	For non-resectable parotid cancer	Mainly cohort studies, a few non-randomized controlled trials
Chemotherapy, adjuvant	No effectivity is adjuvant therapy together with radiotherapy, compared to adjuvant therapy alone	Cohort studies
Chemotherapy, palliative	Low effectivity	Descriptive studies
Biologicals	No clear demonstration of effectivity in metastatic/recurrent parotid cancer	Small controlled non-randomized phase I-II trials

## Methods

A literature search was conducted using the PubMed database with the following MeSH terms (including subheadings): “parotid gland”, “parotid neoplasm”, “cancer of parotid”, and “facial nerve” (last search on 31-December-2017). A total of 183 articles were finally included into the analysis, based on relevance, scientific evidence and actuality. If possible, studies of the last ten years were preferably included. Studies investigating therapy of parotid tumors within prospective or even randomized controlled trials are very rare due to the rarity of the disease and the diverse histologies leading to a lack of high level evidence. If presented recommendations are based on studies with high level of evidence, data are presented in more detail.

## Results

### Epidemiology

Epidemiological data on benign tumors are sparse. Pleomorphic adenoma is the most common tumor of all salivary glands with a reported incidence of 2.4–4.9/100,000 persons/year, and constitutes 40–60% of parotid tumors (9–11). Warthin tumor is the second most common tumor with a frequency of about 30%. A relative increase of the Warthin tumors in the recent years has been observed (12), probably due to the increasing ageing of humankind. This might have implications on the therapy strategies (see below). The term cystadenolymphoma for a Warthin tumor should not be used anymore to avoid confusion with a lymphoma. All other types of benign tumors are very rare.

The annual incidence of epithelial malignant tumors of the major and minor salivary glands varies between 0.3-3/100,000 persons/year. Due to population-based studies on parotid cancer from different countries, the frequency of the most important phenotypes is variable: acinic cell carcinoma (10–18%), adenoid cystic (9–15%), adenocarcinoma (13–32%), mucoepidermoid carcinoma (11–31%) and carcinoma ex pleomorphic adenoma (7–13%), squamous cell carcinoma (9–17%), salivary duct carcinoma (3–6%) (4, 13, 14). The reasons for this variability are unknown. With the proviso that a relevant misclassification is excluded, the variability of phenotypes explains different survival rates (see below) for parotid cancer in different populations.

### Basic Diagnostics for Patients with a Parotid Tumor

Most tumors present as a palpable and clearly delimitable lump. Pain, rapid growth, facial palsy, or fixation to the surrounding tissue, are typical signs of malignancy. Most tumors are located in the superficial lobe. The superficial lobe is readily visualized with high frequency ultrasound (US) (15). Like for any imaging method the specificity of ultrasound in assessment of the histology of a tumor is low (16). It has to be emphasized that the accuracy of CT and MRI is not better but these cross-sectional imaging techniques are much more expensive and less quick available. Furthermore, US can be used to guide fine-needle aspiration cytology (FNAC). This unique combination of US and FNAC has a high level of diagnostic accuracy and safety (17). The main trunk of the facial nerve normally cannot be visualized by standard US machines but the same holds true for CT and MRI. Only the position of the tumor relative to the retromandibular vein gives hints for the localization of the tumor in the superficial or the deep lobe.

US is operator-dependent. It is frequently used for diagnostics of parotid tumors in countries where US is performed by the head and neck surgeons themselves. The surgeon can combine the clinical examination directly with the US examination without any time delay (18). Otherwise, the patient has to be seen by a consultant radiologist with a subspecialty interest in head and neck imaging. In such a work-flow US loses of its benefits. Surgeons who do not perform US by themselves are more familiar with MRI or CT images (17, 19). Hence, national medical training structures are influencing the work-flow for parotid tumors much more than the accuracy of the imaging technique.

### Fine-Needle Aspiration Cytology Is a Basic Diagnostic Tool for All Parotid Tumors

Fine-needle aspiration cytology (FNAC) has become a standard diagnostic test in the initial evaluation of a parotid mass in many places. Typically, 25-gauge needles are used for FNAC and sometimes 22-gauge needles are required for deeper lesions. The method is easy to learn, has a low complication rate, and can be performed quickly by the head and neck surgeon himself. The risk for bleeding and hematoma formation seems not be increased in patients taking antithrombotic or anticoagulant medications (20).There is no evidence for tumor seeding after FNAC. Several meta-analyses have confirmed the high overall diagnostic accuracy of FNAC. In the latest meta-analysis of including data from 6784 FNACs, FNAC had a 96% (95% CI [95% CI] 94–97%) overall diagnostic accuracy in distinguishing benign from malignant parotid tumors (21). Among the subgroup of prospective trials and using the combination with ultrasound guidance, the pooled analysis demonstrated a sensitivity of 88.2% (95% CI, 50.9–98.2%) and a specificity of 99.5% (95% CI, 96.0–99.9%). The probabilities of non-diagnostic and indeterminate cytology were 5.3% (95% CI, 3.0–7.5%) and 14.7% (95% CI, 10.6–18.8%), respectively. It is very important that there was significant heterogeneity found among studies (72.4% for sensitivity and 78.6% for specificity). The underlying reason might be the lack of a universal classification system for the cytologic evaluation and reporting of parotid gland lesions. Therefore, the American Society of Cytopathology and the International Academy of Cytology have proposed a unified system (Milan system) for reporting of FNAC of salivary gland lesions (22). FNAC is also accurate for the diagnosis of secondary malignancies in the parotid gland, for instance for metastasis of skin cancer including melanoma or metastatic squamous cell carcinoma from other sites than the skin (23).

Although the good accuracy of FNAC has been proven in several trials and meta-analyses, some otolaryngologists and hospitals still refuse FNAC. The underlying reasons seem not to be of scientific but again of structural nature. It might be that adequate clinical skills for ultrasound examinations needing for better FNAC guidance are missing or that a skilled salivary gland cytologist is missing.

The combination of a thorough clinical, sonographic and cytologic evaluation at the initial visit enables a rapid workup of the patient. The surgeon has the benefit of real-time imaging and direct correlation with history and physical exam findings, making assessment a dynamic process (19). In case of a cytologically confirmed benign parotid tumor and benign US features and no obvious deep lobe extension, US alone provides enough imaging information for the surgeon before parotid surgery.

### How to Deal with an Inflamed Parotid Tumor

Pain can occur when a benign tumor is inflamed. This is mostly seen for Warthin’s tumor (24). Inflammation can lead to poorly defined lesion margins in sonography or MRI, which may be misinterpreted as signs of malignancy. In cases of clinical signs of acute inflammation, the patient should be treated with antibiotics and analgetics. Serological examinations might be necessary in selected cases. The patient should be reevaluated not later than 72 h after initial diagnosis. If symptoms were related to an acute inflammation, the symptoms should improve within this time interval. A clinical and ultrasound control is scheduled 2–3 weeks later and surgery is planned after full recovery of the inflammation. In contrast, if the symptoms persist or deteriorate, and further MRI diagnostics are suspicious for malignancy, the tumor should be handled like parotid cancer until proof for the contrary is provided.

### Extended Diagnostics for Patients with a Benign Parotid Tumor

The deep lobe of the parotid gland is difficult to evaluate by US because it is obscured by the ramus of the mandible. A MRI is method of choice when there is sonographic suspicion of deep lobe involvement. MRI should be preferred over CT because of its superior contrast, better spatial resolution, and lack of radiation. CT should be used if MRI is not available.

### When Is a Core Needle Biopsy Helpful?

An open excisional biopsy as alternative to FNAC cannot be recommended because of the risk of tumor spillage, facial nerve injury, scarring, and fistula formation (21). The question is if an ultrasound-guided core needle biopsy (CNB) with an 18-gauge needle poses an alternative to FNAC, especially if the FNAC result is inconclusive. Any CNB entails the risk of tumor seeding along the needle tract and the risk seems to depend on the needle size. Studies investigating rates of tumor recurrences in dependence of the needle diameter are lacking (25). Of course, and in contrast to FNAC, a CNB obtains a tissue conglomerate allowing a histopathological diagnosis including immunohistology. Like for FNAC, ultrasound guidance seems also to improve the accuracy of CNB. A recent meta-analysis revealed a sensitivity of 96% (95% CI, 87–99%) and a specificity of 100% (95% CI, 84–100%) for ultrasound guided CNB (25). The disadvantages of CNB are that it needs local anesthesia, is more painful, and is associated with a higher incidence of hematoma (up to 1.6%) and facial nerve injury (about 0.2%) (21). The variation of accuracy between hospitals seems to be lower for CNB than for FNAC (25). CNB might be an alternative in hospitals where FNAC cannot be established or contrasts with the clinical presentation. Another strategy is to use CNB as an option for selected cases when the cytopathologist suggests after initial review of a FNAC that a definitive diagnosis requires a more extensive tissue sample (26).

### Dealing with Parotid Gland Incidentalomas

As a result of more frequent application of PET/computed tomography (PET/CT) for staging and follow-up of various malignancies, there are more PET/CT reports about abnormal 18F-FDG uptake in unexpected locations such like the parotid gland (27). FDG-positive parotid incidentalomas occur in less than 14% of PET/CT scans, of which only 4% are malignant. Warthin’s tumor seems to be the most frequent histology of parotid incidentalomas and show the highest mean standardized uptake value (SUV_max_). Nevertheless, PET/CT is unable to differentiate benign from malignant parotid lesions based on SUV_max_ alone (27). Due to the low frequency of malignant incidentalomas, their management can be performed as for any benign parotid tumor, i.e., FNAC is recommended as a next step. However, there is one exception: patients with a prior history of lymphoma appear to be at a much higher risk of parotid malignancy and warrant in any case a further investigation (28).

### Special Situation: It Looks Like a Warthin Tumor – Is Wait and Scan Allowed?

Even the best imaging and optimal FNAC can never completely rule out a parotid malignancy. Then again, the accuracy of FNAC in centers using FNAC routinely is extraordinarily high for Warthin tumor and the risk of malignant transformation seems to be very low for this benign tumor type (29). Warthin tumors seem to have a wide range of growth rates. In a small series of 13 patients who underwent observation with serial CT or MRI, the average growth per diameter was 8% per year (95% CI −27 to 43%; range −148 to 460%; median −8%), and was highly variable (SD 96%)(30). Hence, the growth rate cannot be used for decision making for or against conservative management. Warthin tumor is often seen in elderly patients (12). Especially the amount of patients with Warthin tumor and higher comorbidity leading to higher risks for surgery under general anesthesia is thereby increasing. A conservative management might be reasonable for patients with a confident diagnosis of Warthin’s tumor on FNAC, an accurate history, clinical examination, and imaging (29). If such a protocol is followed, the patient must be instructed on recognizing the signs and symptoms of malignancy or malignant transformation so that he can seek immediate evaluation. This might be difficult or impossible for some elderly patients. Regular follow-up with interval ultrasound examinations are an imperative part of a conservative strategy.

### Extended Diagnostics for Patients with Parotid Cancer

MRI is the method of choice for patients with palpable masses and suspicion of a malignant tumor (31, 32). MRI provides information on the exact localization and extent of the lesion, also in the deep lobe and the parapharyngeal space. MRI allows detecting perineural spread, bone invasion and meningeal infiltration (32). These and deep infiltration into the parapharyngeal space are signs of malignancy. These findings are not observed in benign lesions. Like for extended diagnostics for benign tumors, CT should be used for further staging if MRI is not available or contraindicated. Fluorine-18 FDG PET/CT is not helpful to differentiate benign, malignant, and metastatic parotid tumors (33). PET/CT parameters, including total lesion glycolysis, metabolic tumor volume, standardized added metabolic activity, and normalized standardized added metabolic activity, are not able to differentiate benign from malignant parotid tumors, primary parotid tumors from metastasis, or metastasis from squamous cell carcinoma and non-squamous cell carcinoma metastasis. There is only sparse data indicating that PET/CT is more sensitive than other imaging tools to detect occult cervical lymph node metastasis (34, 35). All malignant tumors of the parotid gland are classified according to the current edition of the Union International Contra Cancer/American Joint Committee (UICC/AJCC) cancer staging system (Tables 2, 3).

**Table 2:** TNM Classification of parotid cancer.

**T - Primary tumor (identical for cT and pT)**	
TX	Primary tumor cannot be assessed
T0	No evidence of primary tumor
T1	Tumor 2 cm or less in greatest dimension without extraparenchymal extension*
T2	Tumor more than 2 cm but not more than 4 cm in greatest dimension without extraparenchymal extension*
T3	Tumor more than 4 cm and/or tumor with extraparenchymal extension*
T4a	Tumor invades skin, mandible, ear canal, and/or facial nerve
T4b	Tumor invades base of skull, and/or pterygoid plates, and/or encases carotid artery
**cN - Regional Lymph Nodes**	
N1	Metastasis in a single ipsilateral lymph node, 3 cm or less in greatest dimension without extranodal extension (ENE-)
N2a	Metastasis in a single ipsilateral lymph node, more than 3 cm but not more than 6 cm in greatest dimension without extranodal extension (ENE-)
N2b	Metastasis in multiple ipsilateral lymph nodes, none more than 6 cm in greatest dimension, without extranodal extension (ENE-)
N2c	Metastasis in bilateral or contralateral lymph nodes, none more than 6 cm in greatest dimension, without extranodal extension (ENE-)
N3a	Metastasis in a lymph node more than 6 cm in greatest dimension without extranodal extension (ENE-)
N3b	Metastasis in a single or multiple lymph nodes with clinical extranodal extension (ENE+)**
**pN - Regional Lymph Nodes**	
pNX	Regional lymph nodes cannot be assessed
pN0	No regional lymph node metastasis
pN1	Metastasis in a single ipsilateral lymph node, 3 cm or less in greatest dimension without extranodal extension (ENE−)
pN2a	Metastasis in a single ipsilateral lymph node, less than 3 cm in greatest dimension with extranodal extension (ENE+) or, more than 3 cm but not more than 6 cm in greatest dimension without extranodal extension (ENE−)
pN2b	Metastasis in multiple ipsilateral lymph nodes, none more than 6 cm in greatest dimension, without extranodal extension (ENE−)
pN2c	Metastasis in bilateral or contralateral lymph nodes, none more than 6 cm in greatest dimension, without extranodal extension (ENE−)
pN3a	Metastasis in a lymph node more than 6 cm in greatest dimension without extranodal extension (ENE−)
pN3b	Metastasis in a lymph node more than 3 cm in greatest dimension with extranodal extension (ENE+) or multiple ipsilateral, or any contralateral, or bilateral node(s) with extranodal extension (ENE+)
**M - Distant Metastasis**	
M0	No distant metastasis
M1	Distant metastasis

*Extraparenchymal extension is clinical or macroscopic evidence of invasion of soft tissues or nerve, except those listed under T4a and T4b. Microscopic evidence alone does not constitute extraparenchymal extension for classification purposes.

**The presence of skin involvement or soft tissue invasion with deep fixation/tethering to underlying muscle or adjacent structures or clinical signs of nerve involvement is classified as clinical extra nodal extension (ENE+). Midline nodes are considered ipsilateral nodes.

**Table 3:** TNM Stages for parotid cancer.

Stage 0	Tis N0 M0
Stage I	T1 N0 M0
Stage II	T2 N0 M0
Stage III	T3 N0 M0
	T1, T2, T3 N1 M0
Stage IVA	T1, T2, T3 N2 M0
	T4a N0, N1, N2 M0
Stage IVB	T4b Any N M0
	Any T N3 M0
Stage IVC	Any T Any N M1

### Staging of Intraparotideal Lymph Nodes and Cervical Lymph Nodes

Sonography and cross-sectional imaging are also needed for locoregional nodal staging. The parotid gland itself contains about 20 lymph nodes. Beyond the intraparotid nodes, there are the parapharyngeal and retropharyngeal nodal groups that have not been included in the UICC/AJCC cancer staging of the neck in primary parotid cancer. This is important to notice because a patient, for instance, with an intraparotid metastasis is classified as *N*+ in the same manner as a patient with a neck metastasis.

Analogous to other head and neck cancer subsites, cervical lymph node metastasis (and in case of parotid cancer also intraparotideal lymph node metastasis) is one of the major prognostic factors determining patient survival (36). The awareness of the special role of intraparotid metastatic lymph nodes originally came up from cutaneous skin cancer. The parotid lymph nodes represent an important group of nodes at risk for metastatic involvement from cutaneous malignancies of the head and neck (37). The overall rate of cervical lymph node metastasis (pN+) amounts to 24–53%. The rate of intraparotid lymph node metastasis (pP+) is about 39% (38). If neck metastasis occurs, often more than 3 neck levels are involved (36). Occult metastasis (cN0/pN+) is about 12–45%, i.e., the rate of occult metastasis is high (39, 40). Occult metastasis can occur not only in the neck but also in the intraparotid lymph nodes (cP−/pP+) with a rate of up to 30% (36, 40, 41). These numbers guide to a more aggressive therapeutic approach at the primary site (to include as much as possible of the intraparotid lymph nodes) and at the neck (low barrier for the indication of neck dissection or neck irradiation, see below). Furthermore, the surgeon should ask his pathologist to routinely look for intraparotid lymph nodes in the parotid specimen.

### The Role of Histopathology of Parotid Tumors

The new WHO classification from 2017 for the first time describes less malignant tumor entities than the last edition (Table 4) (42). Nevertheless, the 4th edition from 2017 still describes 20 different malignant phenotypes. Due to the constant changes of the classification system, it is important to notice in publications which edition was used and if older diagnoses were re-classified or not. Reclassification of parotid cancer can lead to a significant amount of classification changes with impact on prognostic predictions based on phenotypes (44). Better understanding of the molecular pathogenesis, especially of defining translocations and gene fusions, are powerful diagnostic tools to define the phenotype and has much influenced the new classification (43, 45). The most important clinical message still is that histologic overlap between many entities, including both benign and malignant neoplasms, considerably adds to the diagnostic challenge for surgical pathologists (46). In doubt and in case of the diagnosis of a very rare tumor, catching a second opinion is recommended. Second, it is important for the clinician to notice that the classification for low-grade and high-grade tumors is only defined for some entities. Therefore, decision-making for adjuvant therapy (see below) after primary surgery based on this criterion is not possible for all parotid cancer types. The malignant phenotype or its molecular characteristics have so far no influence on the therapy strategy in clinical routine, with one exception: in adenoid cystic carcinoma, postoperative radiotherapy (see below) is normally always recommended independent of the tumor stage.

**Table 4:** WHO histological classification of salivary gland tumors 2017 (42, 43).

**Benign epithelial tumors**	**Malignant epithelial tumors**
Pleomorphic adenoma 8940/0	Acinic cell carcinoma 8550/3
Myoepithelioma 8982/0	Mucoepidermoid carcinoma 8430/3
Basal cell adenoma 8147/0	Adenoid cystic carcinoma 8200/3
Warthin tumor 8561/0	Polymorphous adenocarcinoma 8525/3
Oncocytoma 8290/0	Epithelial-myoepithelial carcinoma 8562/3
Canalicular adenoma and other ductal adenomas 8149/0	Clear cell carcinoma 8310/3
Sebaceous adenoma 8410/0	Basal cell adenocarcinoma 8147/3
Lymphadenoma	Sebaceous adenocarcinoma 8410/3
Sebaceous 8410/0	Secretory carcinoma 8502/3
Non-sebaceous 8410/0	
Ductal papillomas 8503/0	Intraductal carcinoma
	Oncocytic carcinoma 8290/3
Sialadenoma papilliferum 8406/0	Salivary duct carcinoma 8500/3
Cystadenoma 8440/0	Adenocarcinoma, NOS 8140/3
	Myoepithelial carcinoma 8982/3
**Soft tissue tumors**	Carcinoma ex pleomorphic adenoma 8941/3
Hemangioma 9120/0	Poorly differentiated carcinoma 8020/3
	Carcinosarcoma 8980/3
**Hematolymphoid tumors**	Squamous cell carcinoma 8070/3
Hodgkin lymphoma	
Diffuse large B-cell lymphoma 9680/3	
Extranodal marginal zone B-cell lymphoma 9699/3	Lymphoepithelial carcinoma 8082/3
Hodgkin lymphoma	Sialoblastoma 8974/1
	**Secondary tumors**

### Estimates of the Risk of Malignant Transformation of a Benign Parotid Tumor

There is a lack of epidemiological studies on benign parotid tumors. Text books still spread the believe that up to 25% of benign tumors undergo malignant transformation (47). Actually, only for pleomorphic adenoma the ability to transform into a carcinoma ex pleomorphic adenoma is proven. The true rate of malignant transformation in recurrent pleomorphic adenoma was 3.3 and 3.2%, respectively, in two recent epidemiological studies from Denmark and the Netherlands (10, 11). The transformation occurred at a median time of 5.8 year after initial diagnosis (10). Hence, the risk of malignant transformation is much lower than reported before from uncontrolled small hospital-based series.

### Surgery for Benign Parotid Tumors

Over decades primary parotid surgery for benign tumors by formal parotidectomy has developed into a reproducible, conservative operation with low morbidity. However, there is a tendency to more limited resections in the last two decades. The European Salivary Gland Society has recently published a classification using several levels of dissection (in the style of the neck dissection levels) meeting the needs to better categorize especially the limited parotid resection techniques (Table 5) (48). It is recommended to use this classification, especially in clinical registries and clinical trials. When deciding upon the extent of surgery for benign parotid tumors, the main factors are the location of the tumor, the size of the tumor, and the histological phenotype (49). The classical approach for a lump in the superficial lobe would be a partial parotidectomy (level I or II) or superficial parotidectomy (level I and II;). Most deep lobe tumors still are classically resected by total parotidectomy (level I–IV). In case of small mobile superficial tumors, extracapsular dissection is an alternative for experienced surgeons. In the hands of an experienced surgeon, up to 50–60% of all benign cases might be suitable for extracapsular dissection (50). As extracapsular dissection needs a good selection of appropriate cases, this technique cannot be recommended for non-advanced inexperienced surgeons. There might be an elevated risk of recurrence in hands of novices or if the cases were not well sorted (51–54). Furthermore, the parotid surgeon must be able to switch from extracapsular dissection to a classical parotidectomy technique at any time of surgery. How often such a switch is necessary, is unknown.

**Table 5:** Classification of parotid resection types by the European Salivary Gland Society (48).

Resection	Resected levels and other factors
Superficial superior lobe	I
Superficial inferior lobe	II
Deep inferior lobe	III
Deep superior lobe	IV
Accessory parotid tissue	V
Parotidectomy	Dissection of the facial nerve +resection ≥ 1 level
Formal parotidectomy types	
Lateral parotidectomy	Level I + II
Total parotidectomy	Level I − IV
Partial parotidectomy types	
Partial lateral parotidectomy	Level I or II
Selective deep lobe resection	Level III or IV
Extracapsular dissection	No dissection of the facial nerve and/orResection <1 level

### To Compare Superficial Parotidectomy with Extracapsular Dissection Is to Compare Apples with Oranges

It is not possible to compare directly formal parotidectomy techniques with extracapsular dissection. However, there are even several meta-analyses published comparing both principle techniques [overview in: (49)]. The results of all these meta-analyses are misleading as the patients for extracapsular dissection had selected small mobile superficial tumors whereas the patients who underwent a formal parotidectomy technique represent unselected series of tumors of the superficial lobe. A study comparing both techniques in the same patients’ collective, i.e., with small mobile superficial tumors, (and at best in a prospective trial) does not exist. Both techniques are not competing but complementary techniques in the hand of an experienced surgeon. Table 6 is trying to recapitulate the key characteristics of the techniques side-by-side. Thereby it has once again to be taken into account that prospective trials comparing the different techniques are lacking. It is very important to understand the different philosophy behind superficial parotidectomy and extracapsular dissection: Parotidectomy is facial nerve dissection surgery, i.e., surgery is primarily following the plane of the peripheral facial plexus, whereas extracapsular dissection is following the borders of the tumor without exposing the facial nerve. Therefore, facial nerve monitoring is an absolute must during extracapsular dissection, i.e., a good functioning of the monitoring is essential. Extracapsular dissection implies the removal of a cuff of healthy tissue around the nodule. Partial parotidectomy is something in-between formal parotidectomy and extracapsular dissection: The facial nerve is exposed in the region of the tumor to understand the relation of the nerve to the tumor and then the resection finally follows the tumor. Because the risk of complications (temporary/permanent facial nerve palsy, salivary fistula, Frey’s syndrome) is directly related to the extent of surgery, it seems evident that extracapsular dissection theoretically should have the lowest morbidity. An exception might be the risk of sialocele formation. There is moderate evidence that any resection less than superficial parotidectomy has a higher risk for sialocele formation (55, 56). A very important factor for the evaluation of a parotid surgery technique is the immanent risk of tumor recurrence, mainly for pleomorphic adenoma surgery (52). The risk of recurrence of pleomorphic adenoma is probably forced by their characteristic pseudopodia and areas without pseudocapsule. It is presumed that dissecting along the tumor increases the risk of cutting of or leaving microscopic parts of the tumor *in situ*. This is a classical argument against extracapsular dissection. A typical rebuttal is that even during parotidectomy it is not possible to guarantee a border of normal parotid tissue, namely in areas where the tumor has contact to the facial nerve. Howsoever, due to the long median recurrence time of pleomorphic adenoma, follow-up data at best longer than 15 years are needed for the evaluation of the risk of recurrence. Such long follow-up data of large collectives are only available for formal parotidectomy (more details presented below, subchapter on prognosis).

**Table 6:** Comparison of different surgical techniques to approach to superficial lobe of the parotid gland*

Parameter	Superficial parotidectomy	Partial parotidectomy	Extracapsular dissection
Selection necessary	No	Yes	Yes
Standardized defined approach	Yes	No	No
Experience needed	Low	Moderate	High
Facial nerve monitoring	Recommended	Recommended	Always necessary
Morbidity	Moderate	Low	Low
Long-term data available	High	Moderate	Low

*This is a personal conclusion of the authors based on the literature cited in the text (mainly studies with only moderate evidence) and on their personal experience.

### Special Situation: Localization of the Tumor or Extension Into the Parapharyngeal Space

Salivary gland tumors are the most common type of parapharyngeal space tumors (about 45%). Many of the parotid tumors are asymptomatic and are large when discovered. Due to the neighborhood to the internal carotid artery and several cranial nerves, a good overview about the tumor and its relation to the important anatomical structures of the parapharyngeal space are essential. The transcervical route or the transcervical-transparotid route, i.e., a combination of the transcervical route with a total parotidectomy, are both still the standard approaches. Nowadays, mandibulotomy is rarely necessary (57). Due to the localization and size of the tumors, the tumors are most often pulled out like during extracapsular dissection for tumors of the lateral parotid gland. Nevertheless, a pure extracapsular dissection sparing all other parotid tissue still is experimental (58). The same applies to the endoscope-assisted or a robotic transoral approach. Only casuistic results have been published yet (59).

### Tumor Spillage During Surgery

Tumor spillage during parotid surgery is mainly a problem when dealing with pleomorphic adenoma. Adenomas can be irregularly shaped with a bosselated surface especially in areas with facial nerve contact. Due to their slow and displacing growth behavior, some tumors are under significant pressure. Opening the pseudocapsule of such a tumor *ex vivo* might be followed by an extrusion of the tumor substance. The substance should be suctioned and the open should be sealed with fibrin glue. Rinsing the situs with any type of irrigating solution is controversial. Opponents argue that rinsing might actively force the spreading of viable cells (60). Anyhow, if these measures decrease the risk of recurrence is unclear. The same holds true for adjuvant radiotherapy. There is no clear evidence that adjuvant radiotherapy after spillage reduces the risk of recurrence (60, 61). Probably, most often intraoperative tumor spillage is unnoticed microscopic spillage (62). That microscopic spillage might increase the risk of recurrence seems to be obvious, but is unproven.

### Role of Intraoperative Facial Nerve Monitoring

Routine use of electromyographic (EMG) facial nerve monitoring has increased as an intraoperative adjunctive method during parotid surgery (63). Nevertheless, there is a lack of prospective controlled trials on the efficacy of the EMG monitoring. In a recent meta-analysis of the literature from 1970 to 2014, only 7 out of 1,414 studies fulfilled the quality criteria to be analyzed. Overall, 546 cases of superficial or total parotidectomy were included, mainly (88%) for benign tumors (64). Hence, conclusions for the role of facial nerve monitoring in parotid cancer should be drawn only with caution. This meta-analysis revealed that facial nerve monitoring decreases the risk of immediate but not of final postoperative facial nerve weakness in primary (but not in revision) parotidectomy cases. It is not surprising that the statistics did not reveal an effect on final outcome: We should be aware that temporary facial nerve dysfunction after parotidectomy occurs in 20–40% of patients, but permanent facial nerve weakness only in 0–4% of patients (9, 65–67). All studies performed so far were underpowered to show an effect on final facial function. Facial nerve monitoring also can perform other tasks. It might help to localize the facial nerve and its branches when combining electrostimulation with the monitoring. Thereby, it reduces the surgery time in cases of primary superficial parotidectomy (63). Facial nerve monitoring also helps the surgeon to avoid facial nerve injury when the facial nerve is not exposed during parotid surgery, for instance during extracapsular dissection. Therefore, it is a mandatory requirement when performing an extracapsular dissection. Finally, facial nerve stimulation can be, like in vestibular schwannoma surgery, a prognosticator for facial nerve functional outcome. A low ratio of the maximal amplitude after electrostimulation prior facial nerve dissection versus after the facial nerve dissection is a predictor for postoperative facial nerve dysfunction (68). However, the parotid surgeon should be aware that even 4-channel facial nerve monitoring does not cover the whole hemiface but only a selection of four muscles. Furthermore, a uniform functional allocation of specific peripheral facial branches to a specific mimic movement by active facial nerve stimulation is not possible (69).

### Recurrent Benign Parotid Tumor

Recurrence of a benign tumor occurs mainly as a recurrence of a pleomorphic adenoma. The special features associated with risk of recurrence of pleomorphic adenoma were recently reviewed in this journal (53). Recurrence of a pleomorphic adenoma is most often a multinodular recurrence than a uninodular recurrence (70). The clinical examination and also MRI regularly underestimate the real number of nodules because many nodules have a size of less than 1 mm if the patient occurs with a recurrence. In patients with a first recurrence of a pleomorphic adenoma, especially if first surgery was limited surgery (extracapsular dissection, partial parotidectomy), total parotidectomy is revision surgery of choice. This approach decreases the risk of re-recurrence (71). The morbidity, especially the risk of facial nerve palsy is increased. Treatment of recurrence of other benign phenotypes or re-recurrences of pleomorphic adenoma has to be individualized reaching from wait-and-see policy over enucleation of nodules to radical surgery. Radiotherapy is offered in some countries as a non-surgical alternative for local tumor control but may include an increased risk to induce later a malignant transformation (72). Malignant transformation of a pleomorphic adenoma is a rare event. If it occurs, the prognosis is poor for tumors with extracapsular extension (73).

### Surgery for Parotid Cancer

The extent of parotidectomy is case of a parotid malignancy is still controversial (74). Total parotidectomy is the most frequently used approach for resection of parotid cancer. From an oncological point of view, the deep portion does not differ from the superficial portion apart from its smaller volume with fewer nodes present (75). The deep lobe should be resected to complete the lateral parotidectomy to a total parotidectomy if the primary is located in the deep lobe or has a direct extension into the deep lobe and further, if the tumor has spread to superficial intraparotid nodes or cervical nodes. Finally it is recommended to remove the deep portion for all high grade primary parotid cancers. Conversely, a minority of authors take the view that superficial parotidectomy is sufficient for T1 (or even T2) superficial low-grade tumors if the facial nerve is sufficiently distant from the tumor (76). Hereby, it must be considered that such recommendations are only based on retrospective studies, grading of the tumor is often only determined by final histology, and the distance to the facial nerve is not well defined.

In patients with a normal preoperative facial nerve function and absence of microscopic perineural infiltration, great care has to be taken to preserve the facial nerve. In doubt, a preoperative electromyography can be helpful, as a pathological facial electromyography can indicate a facial nerve infiltration even in patients with clinically normal facial function (77, 78). From an oncological point of view in patients without facial nerve infiltration, sacrifice of parts of the nerve as radical parotidectomy does not offer better tumor control or survival advantage (79, 80). If the facial nerve is infiltrated, a radical parotidectomy is indicated. The parts of the facial nerve which are infiltrated by the tumor are resected.

### When Is It Useful to Take Intraoperative Frozen Sections?

Frozen sections are helpful if FNAC is not available, if it was without clear results, or if there is a discrepancy between the result and the clinical appearance. It should be emphasized that frozen section diagnostics might be difficult in cases where FNAC or a core biopsy had already difficulties (81). Due to a recent meta-analysis including 13 studies with 1,880 cases, frozen section sensitivity, and specificity were 0.90 (95% CI, 0.81–0.94), and 0.99 (95% CI, 0.98–1.00), respectively (82). In specialized centers even the sensitivity can reach 98.5% (83). Furthermore, frozen sections are supportive when nerve resection is needed. Clear margins should be confirmed with frozen sections (78). Thereby, it is important to notice that adenoid cystic carcinoma is characterized by a specific tendency to spread along the facial nerve (and other nerve fibers) (74). It is even possible that the patient still has a normal facial function but the adenoid cystic carcinoma has nevertheless already spread along the facial nerve. Finally, some surgeons perform frozen section of the subdigastric lymph nodes in a cN0 neck and perform a neck dissection in case of regional metastasis in the frozen section material (78).

### Extension of the Neck Dissection in Parotid Cancer

A clear recommendation can be given for a cN +neck. High-grade tumor, advanced T-stage, extraglandular extension of the tumor, or facial palsy, are risk factors for cN+ (14, 84). The most frequently involved neck levels are II, III, and IV (36). Level I and V can also be involved, especially in high-grade tumors (85–87). We should not forget that the phenotype and grading of the tumor is unknown in most cases preoperatively, i.e., when decision making for the extension of the neck dissection is necessary. Taken together, this means that a therapeutic neck dissection should include all levels I–V for all patients, i.e., a (radical) modified neck dissection should be performed.

Much more difficult is to give a clear recommendation for elective neck dissection in case of a cN0 neck. Elective neck dissection has several functions: It should detect occult metastases. It may allow a more accurate staging. If the N0 status is confirmed it thus may avoid irradiation of the neck. Advanced age (>54–60 years), advanced T-stage and high-grade histology are risk factors for occult metastasis. It is recommended to perform an elective neck dissection at least of level I-III, because these levels are mainly involved (87). Some authors also recommend including level IV (85). In reverse, this means that younger patients with early cancer (T1/T2 stage), or low-grade tumors could also have their neck observed. Following this strategy, level V can always be spared for elective neck dissection (86). It has to be emphasized that that exclusively the final histopathological examination clarifies T stage, tumor phenotype and grading. Furthermore, in a series of T1/T2 tumors treated by modified neck dissection an occult metastasis rate of 17% was observed (88). Therefore, some surgeons recommend an elective neck dissection for all cN0 necks (39, 89). Following the same rules, alternatively, an elective neck radiation can be performed (see below).

### A Special Situation: Surgery for Suspected Benign Tumor, but Histology Revealed Parotid Malignancy

Only 25–30% of parotid cancers present with clear signs of malignancy like facial palsy or skin infiltration. All other tumors more or less behave like benign tumors, i.e., are slowly growing, mobile lumps. Therefore, it is remarkable that only a few studies have analyzed if these patients have a worse outcome when the tumors were primarily not treated as malignant tumors. There is one old series of 539 patients after parotidectomy from the last century with a follow-up of 1–8 years (90). 3.7% of the tumors proved surprisingly to be malignant and had no worse outcome than the benign tumors. In a recent series of 821 patients and a median follow-up of 12 years, 5% of the clinically benign cases were subsequently revealed as malignant histologies (91). The patients were treated by superficial parotidectomy or extracapsular dissection. In the groups of patients treated with extracapsular dissection or superficial parotidectomy, 71 and 53% cases, respectively, had positive or close margins. These patients and patients with high-grade tumors or adenoid-cystic carcinoma received postoperative radiotherapy but no completing revision surgery. Following this concept, the outcome of the patients was as good as for the benign cases in this series (100% 5 year survival). In another recent series treated exclusively with extracapsular dissection. Herein, 19% of all malignant cases were patients with unexpected detection of a malignancy (92). 86% of these patients underwent revisions surgery by total parotidectomy. Residual cancer tissue was detected in 14% of the cases. These patients received adjuvant radiotherapy. The tumor control rate was 96% and the 5 year survival rate was 100%. The series was updated recently for the patients with low-grade malignancies who received only extracapsular dissection without further surgery or radiotherapy (rejected by the patients) (93). Within the median follow-up of 66 months (20–126 months) all patients were tumor-free. As larger series with long follow-up are missing, these data have to be interpreted with caution. From an oncological and conservative point of view, revision surgery in form of a total parotidectomy and selective neck dissection has to be recommended for all other patients with close or positive margins.

### In Case of Facial Palsy: If Possible Single Stage Facial Nerve Reconstruction Surgery

Immediate reconstruction of the facial nerve directly after radical parotidectomy gives the best functional results. The same rule holds true for iatrogenic lesions during surgery of benign lesions. In patients with parotid cancer, the peripheral facial nerve is most frequently infiltrated in its intraparotid facial plexus. Therefore, more or less complex segmental defects occur after radical parotidectomy. Here is no place to describe facial nerve reconstruction in detail. Reference is made to recent overviews (94, 95). Briefly, the reconstruction is performed with an interpositional graft for simple segmental defects, or by hypoglossal-facial jump anastomosis or masseteric nerve transposition, or by combinations of several techniques for complex defects or if the central facial nerve stump is not available or was resected highly up proximal into the mastoid segment. Postoperative radiotherapy is no argument against early reconstruction as the results are finally as good as in other patients. Typically, facial nerve reconstruction surgery is accompanied by upper lid weighting for immediate protection of the eye (96). If nerve reconstruction is not possible or intended due to restricted life expectancy, local muscle transpositions or static sling techniques are methods of choice.

### Radiotherapy: Mainly as Adjuvant Treatment for Parotid Malignancies

In malignancies of any histology, postoperative radiotherapy (RT) is indicated for advanced-stage disease (T3/T4), intermediate or high-grade tumors, close or positive margins, and lymph node metastases, Bone invasion, , perineural and vascular invasion are additional adverse features (7, 97). Several large scale retrospective analyses confirm the impact of adjuvant radiotherapy in improving local control and overall survival (98, 99). Among those the largest analysis is based on the Dutch Head and Neck Oncology group database investigating 498 cases of salivary gland carcinoma in any location (100). Adjuvant RT significantly enhanced local control rates in T3/T4 tumors, perineural and/or bone invasion and close or positive margins. Safdieh et al. recently investigated the role of adjuvant radiotherapy in patients with malignant salivary gland tumors (91.4% located in the parotid gland) as registered in the US National Cancer Database (NCDB) between 2004 and 2012 (8). 4068 patients were included in the analysis (pT1-4 Nx/N0/N1cM0 high grade or pT1-4N1M0 low grade), of which 2728 received postoperative radiotherapy but 1340 did not. Of note, salivary ductal carcinoma and adenoid-cystic carcinoma were excluded. The median radiation dose was 60 Gy in patients with negative margins and 66 Gy in case of positive margins with intensity modulated techniques being increasingly used over the observation period. In multivariate analysis, adjuvant RT significantly improved overall survival (HR, 0.78; 95% CI, 0.71–0.86; *P* < 0.00). Radiation utilization was associated with improved survival in sub-group analyses excluding squamous cells histology and limiting the analysis to patients ≤ 65 years of age. Similarly, a multivariate analysis based on the Surveillance, Epidemiology, and End Results (SEER) data found in 2170 parotid malignancies a survival benefit associated with adjuvant RT (HR, 1.20; 95% CI, 0.98–1.47; *P* = 0.08) (101).

In adenoid-cystic carcinoma, adjuvant RT is indicated in T3/T4 tumors and in T1/T2 tumors even if completely resected if adverse histological features exist, especially perineural invasion (7, 102). In this unique entity, adjuvant RT was associated with improved local control and survival rates in several large retrospective cohorts , e.g., in a cohort of 1784 patients registered in the NCDB and in a recent Japanese retrospective multicenter analysis including 103 patients with adenoid-cystic carcinoma of the head and neck (103, 104).

### Radiotherapy as Definitive Treatment for Parotid Malignancies

In unresectable parotid cancer e.g., cases with involvement of the base of the skull or inoperable cases definitive radiotherapy is indicated. Actually, mostly retrospective data with long intervals of recruitment are available showing a 5 year overall local control rate of about 57–70% (105, 106).

### Technical and Radiobiological Considerations

Image guided, intensity modulated radiotherapy (IMRT) is nowadays the modern standard technique for radiotherapy of parotid cancer (107) for most histologies. In patients with cN0 necks, who have not received elective neck dissection, RT of the neck should be included (101). Given the growth characteristics and the relative radioresistance of this entity, adequately large treatment volumes and sufficiently high radiation dosage determine treatment outcome (104, 108). The known relative radioresistance of salivary gland tumors, especially adenoid-cystic carcinoma led to investigation of radiation qualities with high linear energy transfer (LET) properties. Fast neutron radiotherapy was historically the first high-LET irradiation to be evaluated.(109). Using techniques, that were standard at that time, fast neutron therapy resulted in good 5 year locoregional control rates of 60–80%, even in very advanced unresectable tumors (110) proving the principle of high-LET-effects to overcome radioresistance. However, given the high biologically effective dose (BED) necessary for cure, neutron radiotherapy led to significant late side effects, like dysphagia, pain, osteoradionecrosis, ageusia, and especially trismus (111). In an analysis of 335 patients with very advanced stage malignant salivary gland tumors (211 of 334 cases with T4 tumors, most of them macroscopic residual disease) the rate of late side effects grade ≥3 was 8.9% with a loco-regional control rate of 60.6% (112). The frequency of these side effects can possibly be reduced with radiotherapy using ions with high-LET properties, e.g., carbon ions, whose depth dose distribution facilitates high dose deposition to be restricted to the target volume through steep dose gradients and missing exit dose. This technique shows 5-year local control rates of 59–80% even in advanced or recurrent adenoid-cystic as well as non-adenoid-cystic salivary gland tumors with acceptable toxicity (113). Combined intensity modulated radiotherapy and carbon ion boost resulted in superior locoregional control rate (5 year local control rate: 59.6%) and overall survival rate (5 year overall survival: 76.5%) in a group of 95 patients with very advanced adenoid-cystic carcinoma as compared to photon radiotherapy (5 year local control rate: 39.2%, 5 year overall survival: 58.7%) (108, 114). In a prospective phase II trial investigating intensity modulated radiotherapy combined with dose escalated carbon ion boost in adenoid-cystic carcinoma of the head and neck region this treatment was adequately tolerated with a local control rate of 81.9% and overall survival 78.4% after 3 years (115). With a 5 year local control rate of 81.5% in patients with high-grade malignant non-adenoid cystic carcinoma of the salivary glands, IMRT with boost of carbon ions or definitive carbon ion radiotherapy is an interesting option for these entities as well (113, 116).

### The Limited Role of Chemotherapy for Parotid Cancer

Chemotherapy does not play any role in standard curative treatment of primary parotid cancer. It has to be emphasized that salivary gland cancer was excluded from the milestone trials investigating adjuvant cisplatin-based chemoradiotherapy in squamous cell cancer of the head and neck region (117). Interestingly, when analyzing data from cancer registries, up to 10–17% of patients with salivary gland cancer receive adjuvant radiochemotherapy (4, 118, 119). These patients showed lower survival rates but higher treatment-related morbidity than patients only receiving adjuvant radiotherapy. Probably, some uncontrolled negative risk factors influenced the decision for adjuvant radiochemotherapy (107, 120). Anyhow, a recommendation for radiochemotherapy even for selected cases cannot be deduced from epidemiological data. Hospital-based data is also mainly retrospectively collected and biased as patients with negative risk factors probably had a higher chance to receive adjuvant radiochemotherapy, mainly with platinum-based regimes (121). At present, addition of chemotherapy to adjuvant radiotherapy does not seem to influence survival in comparison to radiotherapy alone, also not for the subgroup of patients with adenoid-cystic carcinoma (122).

### Biologicals for Targeted Therapy of Parotid Cancer Have Not Yet Reached the Clinical Routine Beyond Clinical Trials

More and more information is gained about key molecular alterations and signaling pathways in salivary gland tumors, which can be used for better phenotyping of the tumors (43, 123). Mutation analyses can also be used for outcome prediction (124). Clinical trials using targeted therapy are sparse, restricted at best to phase II trials with small case numbers, and none of the targeted therapies tested so far has shown any convincing antitumor activity (125, 126).

Although neither biological for targeted therapy nor classical chemotherapy, androgen deprivation therapy has to be mentioned here. There is first moderate evidence that patients with androgen receptor positive salivary duct carcinoma, known to be a phenotype with bad prognosis, seem to profit from an androgen deprivation therapy (127).

### Inoperable, Metastatic or Recurrent Parotid Cancer

Recurrent parotid cancer is often highly aggressive with high risk of distant metastases (128). Salvage surgery is restricted to selected cases with limited local or regional recurrence. The most common site of distant metastasis is the lung (85). With regard of distant metastasis, adenoid cystic carcinoma is a special parotid tumor. Delayed hematogenous metastases occur in 25–50% of patients. Second, patients with metastatic adenoid cystic carcinoma, different to patients with metastatic parotid cancer of other phenotypes, can still have a long-term survival. Therefore, lung metastasectomy should be considered as a therapeutic option to achieve local control of disease if complete surgical resection of the metastases is feasible and the time to pulmonary relapse after primary tumor treatment is greater than 36 months (129). There is no standard for palliative chemotherapy for metastatic or unresectable recurrent parotid cancer. Randomized trials have not been performed. There is low evidence for platinum-based chemotherapy [Overview about monotherapy and combination chemotherapy regimes in: (130)]. Effectivity of second-line chemotherapy is more than doubtful. Re-irradiation might be an alternative to palliative chemotherapy. Data on re-irradiation or brachytherapy of parotid cancer are sparse and limited to small case series (131, 132).

### Secondary Metastasis Within the Parotid Gland Is Mainly Head and Neck Skin Cancer

Secondary metastasis of the parotid gland mainly arises from supraclavicular and less frequently form distant infraclavicular primaries (not discussed here). Nodal metastases of cutaneous squamous cell carcinoma to the parotid gland are by far the most frequent secondary metastasis within the parotid gland and the first differential diagnosis when histopathology reveals a squamous cell carcinoma in the parotid gland. About 15% of advanced (≥T2) cutaneous squamous cell carcinomas metastasize to regional lymph nodes in the parotid gland and/or neck (133). The most common location for the primary cutaneous cancer is ear/preauricular (about 39%), temple (19%), cheek (10%), scalp (10%), neck (7%), forehead (4%), lips (3%), and periorbital skin (2%). If there is a parotid metastasis of cutaneous squamous cell carcinoma, about half of the patients have several intraparotid metastases (133). Further on, about two thirds of these patients show simultaneously a clinical negative neck (cN0 in the neck levels beyond the parotid) and one third a clinical positive neck (cN+). Level II is most frequently involved. However, positive nodes are found in all neck levels after therapeutic neck dissection. The same holds true when performing elective neck dissection in a clinically negative neck, i.e., occult metastases are found predominantly in level II, but can be found in all other levels. Occult neck disease is found in about 17% of patients with metastatic squamous cell carcinoma to the parotid gland with higher risk for patients with immunosuppression (134). Due to the distribution of involved nodes it is recommended that a patient with parotid involvement should receive a total parotidectomy (75, 135). In contrast, there is no role for elective parotidectomy even in advanced squamous cell carcinoma of the head and neck skin in absence of clinical disease in the parotid gland (136). In case of a clinically negative neck the patient receives at least a selective neck dissection of level Ib-III. In the case of a posterior primary, level V should be dissected as well. In a clinically positive neck, a modified radical neck dissection is recommended as therapeutic neck dissection (137).

Data on malignant melanoma and Merkel cell carcinoma with metastasis to the parotid gland are sparse. The spreading patterns from the different head and neck skin sites to the parotid and neck levels seem to be similar to cutaneous squamous cell carcinoma. Therefore, the same therapeutic strategies as for cutaneous squamous cell carcinoma are also recommended for metastatic malignant melanoma and Merkel cell carcinoma, especially also when sentinel lymph nodes are found within the parotid gland (138).

### Parotid Tumors in Children

About 5% of all salivary gland tumors occur in children, mainly in the parotid gland. About half of the tumors or even less are benign (139). Half of the benign tumors are non-epithelial tumors like angiomas occurring in early childhood. The other half is epithelial tumors, of which more than 90% are pleomorphic adenomas. These tumors are mainly diagnosed with a mean age of 10–15 years (140). The surgical approach is far more conservative than in adults. Depending on the tumor localization and tumor size, a superficial or total parotidectomy is recommended. Limited approaches like extracapsular dissection cannot be recommended as routine approach. Only case reports with a small number of pediatric patients who underwent extracapsular dissection for a benign tumor are published (141).

Parotid cancer in children is very rare. A salivary gland epithelial neoplasm presenting in a child has a higher probability of malignancy (40–60%) than in adults. Mucoepidermoid carcinoma (45–50%) and acinic cell carcinoma (25–35%) are the most frequent malignant phenotypes in children (139, 142). The therapy concepts do not differ from adult patients (see above) with respect to surgical management of the primary tumor and neck metastasis as well as for the adjuvant radiotherapy. The necessity of elective neck dissection for pediatric parotid cancer remains speculative, as most tumors are of low-grade histology, and occult metastases are rare (143). Some authors recommend elective neck dissection for children with high-grade and advanced stage tumors. If a negative neck is confirmed (pN0) radiotherapy of the neck may be avoided (144).

### Outcome, Prognosis, and Follow-Up of Benign Parotid Tumors

Surgery for benign parotid tumors has reached high standards. Overall, and not differentiating between different surgical techniques, the risk of recurrence of a pleomorphic adenoma after standard surgery is below 4% (53, 145). Due to a recent meta-analysis of 14 studies including 3,194 patients, the mean risk of transient facial palsy, permanent facial palsy, and Frey’s syndrome was 9, 1 and 3%, respectively, for extracapsular dissection and 23, 2 and 19% for superficial parotidectomy (146). Prospective trials systematically analyzing the morbidity and outcome of surgery for benign tumors are still lacking. In a recent prospective trial on 132 patients, the facial palsy rate was 40.2% on the first postoperative day, 28.3% at 2 weeks, 3.9% at 6 months, and 1.6% at 12 months. The overall facial palsy rate was highest for total parotidectomy (54%) lowest for extracapsular dissection (6%) (147) A prospective trial on 66 patients revealed a sialocele rate of 17% and salivary fistula rate of 6%. The risk was higher for extracapsular dissection and partial parotidectomy (55). In another recent prospective trial on 101 patients, the rates of sialocele and salivary fistula were 4.9 and 0.9%, respectively (148). Another long-term prospective trial on 130 patients detected a Frey’s syndrome (asymptomatic and symptomatic) in 46, 46 and 43%, respectively, of patients after 6, 12 and 24 months (149). The same trial revealed a better improvement of the hypoesthesia in the ear region if the posterior branch of the great auricular nerve was preserved. After 12 and 24 months, respectively, 59 and 71% of the patients showed positive sensory test results in the lobule if the nerve branch was preserved, versus 24 and 31% if the nerve branch was not preserved (150). Finally, one recent prospective study on 79 patients used the Facial Disability Index (FDI) as a patient-related outcome measure and the Short-Form 36-Item (SF-36) questionnaire for measurement of general quality of life after superficial parotidectomy (151). Whereas general quality of life was unchanged, physical values on the FDI decreased during the first 3 months and psychosocial values improved significantly from then onwards to 12 months.

### Outcome and Prognostic Factors of Parotid Cancer

The oncological outcome is depending on various patient, tumor, and treatment characteristics. Looking into large hospital-based or population-based studies on parotid cancer outcome, these characteristics are highly variable. This explains why 5 year and 10 year disease-specific survival vary between 55–82% and 47–69%, respectively, between different patients’ collectives [overview in: (152)]. Salivary gland malignancies in children appear to have better clinical outcome, associated with a 10 year disease-specific survival of up to 94% (139). Reflecting the variability in outcome, the prognostic factors detected by univariate analyses are highly variable between different studies. Focusing on multivariate analyses, the best agreement can be found for the following factors: Age, gender, pain, comorbidity, TNM stage, skin invasion, facial palsy, perineural growth, positive surgical margins (R+), and adjuvant radiotherapy. To be able to provide the individual patient with a prognostic estimate, several summary indices (scores, nomograms) have been developed. A highly validated and user-friendly index is the Prognostic Index for Patients with Parotid Carcinoma for pre-treatment and the post-treatment setting (3). The University Hospital Leuven has developed a program for this score (for the use, see http://www.uzleuven.be/parotid). The clinician fills in the prognostic parameters and an output of the software presents the score results and related 5 year recurrence-free probability. Several other nomograms have meanwhile been developed (overview in: (153). The differences are so far marginal. What is needed in the future is the incorporation of molecular characteristics into the indices, as many molecular markers will have an important prognostic impact (124).

## Clinical Guidelines

Recently, the United Kingdom was the first to publish a guideline for the treatment of salivary gland tumors (102). As in this review, the management of benign and malignant tumors is presented in one guideline, taking into account that it is often unclear if the tumor is benign or malignant when the patient presents for the first time or even in some cases until the final histopathology report arrives. Due to the general low to moderate level of evidence, the United Kingdom guideline frequently can only give recommendation based on clinical experience as so called good practice points. The latest update of the U.S. American Clinical Practice Guidelines in Oncology (NCCN Guidelines) for Head and Neck Cancers also provides treatment recommendations for salivary gland cancer (154). These recommendations are formulated in a very general manner and are mainly of low evidence.

### Areas of Uncertainty

Due to the paucity of data, especially due to the scarce number of prospective trials, there are many important areas of uncertainty. For diagnostics a much better clinical differentiation between benign and malignant tumors would be desirable. Contrast-enhanced ultrasound and ultrasound elastography are promising techniques for better differentiation of salivary gland lesions (155). Moreover, a reliable imaging technique showing the facial nerve and its relation to the parotid tumor is still missing. High-resolution ultrasound might overcome this problem (156). The value of FNAC will probably increase when the combination with immunohistochemistry becomes clinical routine (157). Better visualization of the faical nerve during parotid surgery will hopefully decrease facial nerve morbidity even more. First approaches use contrast enhancing optical imaging tool during surgery (158). Very much awaited are the results of the RTOG 1008 trial. This randomized phase II/phase III study is investigating the role of adjuvant concurrent radiation and chemotherapy versus radiation alone in resected high-risk malignant salivary gland tumors (https://www.rtog.org/ClinicalTrials/ProtocolTable/StudyDetails.aspx?study=1008). Finally, it is time for a breakthrough of targeted therapy for parotid cancer.

## Conclusion

Both the clinical and histological diagnostic differentiation between a benign and malignant parotid tumor is challenging. The surgery of benign and malignant parotid tumors has reached a high standard. The actual focus is on less invasive approaches to further reduce the morbidity without reducing the efficacy. Surgery remains the standard of care for resectable parotid cancer. Data on particle radiotherapy increasingly hint at improved local control rates but equal or even reduced toxicity as compared to other high dose radiotherapy techniques. Prospective trials will soon allow establishing the role especially of carbon ion radiotherapy in salivary gland malignancies. Chemotherapy still has no defined place in the treatment of parotid cancer. The breakthrough of molecular targeted therapy also for parotid cancer is urgently expected.

## Ethics Statement

## Author Contributions

All the authors: design of the work; data acquisition, analysis, interpretation, draft contribution, and approval of the final version to be published; agreement to be accountable for all aspects of the work in ensuring that questions related to the accuracy or integrity of any part of the work are appropriately investigated and resolved.

## Conflict of Interest Statement

The authors declare that the research was conducted in the absence of any commercial or financial relationships that could be construed as a potential conflict of interest.
